# Autologous Fat Grafting in the Treatment of Facial Scleroderma

**DOI:** 10.1155/2018/6568016

**Published:** 2018-08-01

**Authors:** Mehdi Gheisari, Arman Ahmadzadeh, Nilofar Nobari, Behzad Iranmanesh, Nikoo Mozafari

**Affiliations:** ^1^Skin Research Center, Shahid Beheshti University of Medical Sciences, Tehran, Iran; ^2^Department of Rheumatology, Shahid Beheshti University of Medical Sciences, Tehran, Iran; ^3^Department of Dermatology, Loghmen-e-Hakim Hospital, Shahid Beheshti University of Medical Sciences, Tehran, Iran

## Abstract

Systemic sclerosis (SSc) is a rare systemic autoimmune disease, characterized by progressive cutaneous and internal organ fibrosis. Orofacial manifestations of systemic sclerosis are extremely disabling and treatment options are limited. In this study, we aimed to assess the safety and efficacy of autologous fat grafting in the face of patients with systemic sclerosis. We enrolled 16 SSc patients suffering from facial sclerosis and limited mouth opening capacity. Autologous fat injection ranging from 15 to 40 ml was administered per patient, based on their face morphology. The patients were evaluated at baseline and 3 months after fat injection. Evaluations included mouth opening capacity, mouth handicap in systemic sclerosis (MHISS), Rodnan skin sclerosis score, skin biophysical properties using a sensitive biometrologic device with the assessment of cutaneous resonance running time (CRRT), volumizing and aesthetic effects based on pre- and posttreatment photographs, possible side effects, and global patient satisfaction. Clinical assessment showed autologous fat transfer significantly improved mouth opening capacity and the MHISS and Rodnan score of patients with facial scleroderma (p value <.001). The aesthetic and/or functional results of fat injection were satisfying to about 80% of the patients. The changes in CRRT values were not significant. Our findings support the possible therapeutic role of autologous fat grafting in improving facial scleroderma both in aesthetic and in functional aspects. This trial is registered with IRCT20180209038677N1.

## 1. Introduction

Systemic sclerosis (SSc) is a rare systemic autoimmune connective tissue disease of unknown etiology, characterized by cutaneous and visceral fibrosis [1]. Key pathogenic abnormality in the skin and internal organs are immunologically overactivated fibroblasts which, with the secretion of extraordinary amounts of collagen and extracellular matrix, lead to progressive cutaneous and internal organ fibrosis [1].

Systemic sclerosis is a heterogeneous disease, but two major clinical subtypes based on the extent of skin involvement are typically recognized, namely, limited cutaneous SSc with skin involvement from the distal to the elbows and knees and diffused cutaneous SSc with skin involvement extending to the proximal limbs and/or trunk [2]. The face is frequently involved in both subtypes of this disease. Patients with facial scleroderma often complain of aesthetic and functional concerns [3]. Facial involvement is associated with disfigurement and limited expression with a mask-like stiffness of the face. The loss of elasticity and the thickening of the skin in the perioral area and lips form perioral radial furrowing and narrowing of the oral aperture, leading to mouth opening reduction that interferes considerably with life's basic functions such as eating, speaking, oral hygiene, and professional dental care [3]. Furthermore, dry mouth or xerostomia because of salivary gland fibrosis and reduced saliva production is also a frequent symptom in these patients that increase the risk of periodontal diseases and caries [4]. The orofacial manifestations of systemic sclerosis are extremely disabling and severely impair the patients' self-image and compromise their quality of life [4].

Autologous fat tissue grafting, in addition to a filling effect, seems to have regenerative potentials presumably due to their adipose-derived stem cells (ASCs) content [5]. Currently, lipotransfer has been used for reversing fibrosis in various conditions such as scars, radio dermatitis, and localized forms of scleroderma such as “en coup de sabre” [6, 7]. More recently, some reports have shown the efficacy of autologous fat grafting in patients with SSc to improve mouth opening and fibrosis reduction in the treated skin [5, 8].

In this study, we aimed to assess the safety and efficacy of autologous fat grafting in the face of patients with systemic sclerosis.

## 2. Material and Methods

This was an open-label study performed at the dermatology operative unit of our center. The study was approved by the ethics committee of the “skin research center”. A written informed consent was obtained from all the participants. All procedures were done free of charge.

We enrolled 16 patients by fulfilling the “American College of Rheumatology” criteria for systemic sclerosis with facial involvement. Exclusion criteria were as follows: (1) pregnant or breastfeeding; (2) patients with diffused skin sclerosis who had insufficient fat for harvest; (3) recently diagnosed patients with SSc who had no clear face and oral involvement; (4) patients with a history of neoplasia in the last 5 years; (5) those who were taking prednisolone of more than 10 mg/d.

### 2.1. Autologous Fat Grafting Procedure

Given an easier access to a sufficient amount of adipose tissues, the trochanteric, the flank, and the periumbilical, or the buttock areas were chosen as the donor sites. The entry points for the infiltration cannula were anesthetized with 1 ml of pure lidocaine with a 30-gauge needle. Then 500 ml of tumescent solution containing normal saline, 25 ml of lidocaine 2%, and .5 ml of epinephrine 1:1000 was infiltrated in the selected donor area with a 1.5 mm cannula. After twenty minutes, the adipose tissue was harvested using a 3 mm blunt tipped cannula connected with a Luer-lock 10 ml syringe, using low vacuum pressure. The collected adipose tissue in each 10 ml syringe was left to sediment by gravity for 10 minutes. Oil and blood excess were eliminated and the remaining fat was transferred to the 1 ml syringe and was directly injected into the face using disposable 18-gauge cannulas.

In this study, 3 of the 4 essential parts of autologous fat transfer technique (donor site preparation, fat harvest, and reinjection) were based on the Coleman method [9]. For adipose tissue processing, the gravity separation technique was used instead of the centrifugation method.

The quantity of injected fat ranged from 15 to 40 ml per patient, based on the morphology of the patients' faces. Subcutaneous injections were administered to different locations: perioral, upper lip, and lower lip, mouth corners, buccal, malar, and periorbital regions. All patients were visited two weeks after AFT to record any possible side effects. During this time, they were asked to contact a physician if any of the following developed: progressive pain, warmness, swelling, and erythema of the face.

### 2.2. Clinical Outcome Evaluations

All patients were evaluated at baseline and 3 months after the procedure. Patient assessment was based on the following:

(1) Mouth opening capacity: maximal distance in cm between the upper and lower incisors.

(2) Mouth handicap in the systemic sclerosis (MHISS) scale, a 12-item questionnaire that specifically quantifies the mouth disability in SSc, is organized in 3 subclasses representing handicap induced by reduced mouth opening (5 items—1, 3, 4, 5, and 6), handicap induced by the sicca syndrome (5 items—2, 7, 8, 9, and 10), and aesthetic concerns (2 items—11 and 12) (Table 1) [10].

(3) Skin sclerosis based on the Rodnan skin score on face (0: uninvolved, 1: mild thickness, 2: moderate thickness, and 3: sever thickness)

(4) Skin biophysical properties: we used a Reviscometer (MPA9; Courage & Khazaka Electronic GmbH, Koln, Germany) to measure the possible changes in the collagen pattern and content. The measurement was based on cutaneous resonance running time (CRRT): the time that acoustical shock waves take to propagate between two sensors (emitter and receiver) on the skin surface. Two sensors are applied to the skin surface in a supine position. The mean CRRT over the four axes (0, 90, 180, and 270) was calculated for the perioral region.

(5) Aesthetic effect: improvement of the patients' appearance was evaluated with the help of pre- and posttreatment photographs. An outside dermatologist was asked to fill out a 4-point scale (-1: worsening, 0: no improvement, 1: some improvement, and 2: much improvement).

(6) Global patients' satisfaction: patients were asked to fill out a 3-point scale to quantify the degree of improvement both from the aesthetic and from the functional points of view (0: unsatisfied, 1: somewhat satisfied, and 2: very satisfied).

### 2.3. Statistical Analysis

All descriptive data are expressed as mean ± standard deviation (SD) or frequency (%). Comparison between values at the baseline and those at 3 months after treatment was performed by paired* t*-test and Wilcoxon's test for continuous and noncontinuous variables respectively. Data analysis was carried out using an SPSS software package version 20 (SPSS Inc., Chicago, IL, USA) and significant levels were considered as P value <0.05.

## 3. Results

### 3.1. Patient Characteristics

Sixteen patients with SSc, all women with a mean age of 39.18 ± 8.32 years, and mean disease duration of 6.5 ± 1.8 years, were enrolled. Six patients were diagnosed with limited SSc and 10 with the diffused form of SSc. The main characteristics of the patients are shown in Table 2.

### 3.2. Side Effects

No serious or persistent complications such as a vascular occlusion phenomenon, fat cyst, facial ecchymosis, or edema developed in the participants. Short lasting adverse effects such as bruising at the zone of fat harvest reported by 10 patients were spontaneously resolved within two weeks. No local or systemic infectious complications related to the procedure were recorded.

### 3.3. Clinical Evaluation of Treatment (Figure 1)

**Figure 2:**
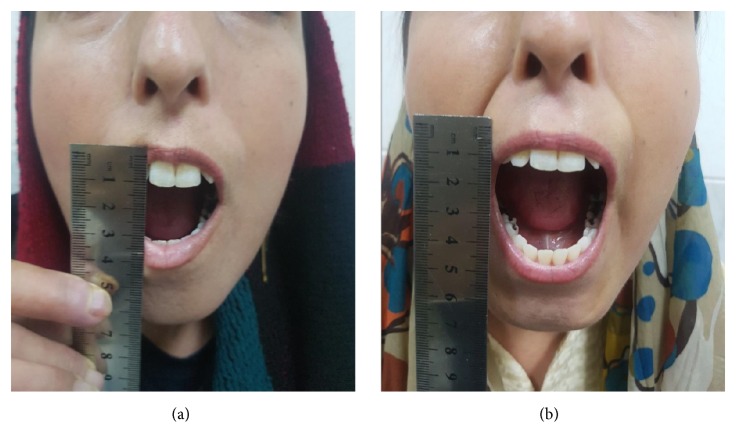
Mouth opening capacity at baseline (a) and significant improvement in mouth opening capacity 3 months after autologous fat transfer (b).

**Figure 3:**
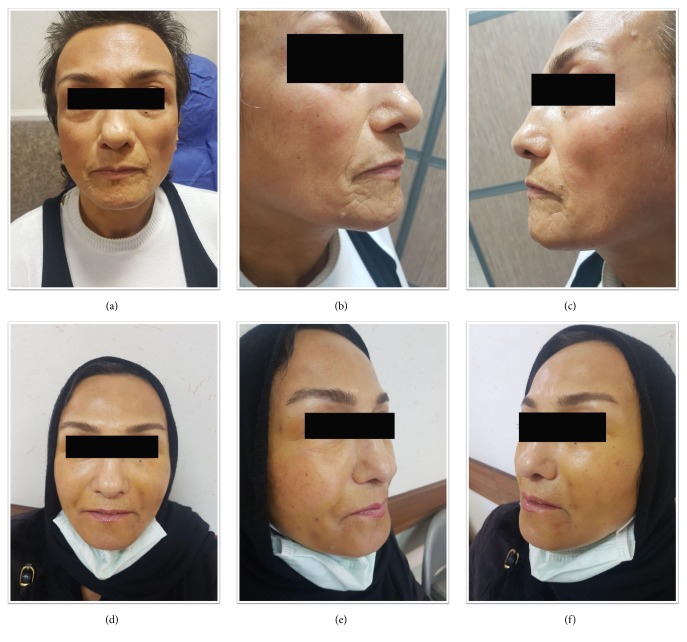
Aesthetic improvement after autologous fat transfer, baseline (a, b, c) and 3 months after autologous fat transfer (d, e, f).

(1) Mouth opening capacity (MOC): in all patients, an improvement in MOC was observed in the 3-month follow-up with a mean gain of .78 cm (range 0.5 to 1.5 cm) (p=<0.001) (Figure 2).

(2) MHISS score: at the baseline, the mean MHISS score was 29.37 ± 4.36 and significantly decreased to 23.25 ± 3.13 after 3 months (mean variation: 6.12 ± 2.3, p=<0.001).

(3) The mean face Rodnan score significantly improved with a reduction from 2.06 ± .57 at baseline to 1.56 ± .51 after 3 months (mean variation: .5 ±. 52, p = 0.001).

(4) The mean CRRT did not significantly change from the baseline (1001.12 ± 369.30) to 3 months after fat transfer (1132.75 ± 315.02) (mean variation: 131.62 ± 150.65, p = 0.39).

(5) Aesthetic effect: 13 of the 16 (81%) patients showed an improvement in their appearance (fuller and softer face with less wrinkles)—no improvement was seen in 3, some improvement in 4, and much improvement in 9 of them (Figure 3).

(6) Global patients' satisfaction: three months after fat grafting, 10 (62.5%) patients said they were very satisfied, 2 (12.5%) patients were somewhat satisfied, and 3 (18.75%) patient were unsatisfied.

## 4. Discussion 

The present study demonstrated that autologous fat transfer (AFT) in the face of patients with scleroderma not only improves facial aesthetic aspects but significantly enhances the mouth opening capacity and reduces skin wrinkles and facial sclerosis. Our study showed that AFT was safe for patients with scleroderma and resulted in the reduction of mouth handicap as assessed by the MHISS score.

In 3 of the 16 patients who were unsatisfied with the aesthetic results, nearly the total volume of injected fat had been absorbed after 3 months, but the improvements in mouth opening and function were retained. There is some evidence to show functional improvement in scleroderma patients following AFT cannot be ascribed only to the filling effects but rather to the activation of various biological mechanisms that could induce tissue regeneration [11, 12].

Recent studies have demonstrated that a fatty tissue has the highest percentage of adult stem cells compared to any other tissue in the body [12]. Adipose-derived stem cells (ASCs), similar to bone-marrow-derived stem cells, are capable of differentiating into multiple mesodermal tissue types, but, in contrast to bone-marrow-derived stem cells, they can be easily harvested by liposuction, and the abundance of these cells (in comparison to bone-marrow-derived stem cells) avoids the need for expansion in culture. Because of these practical aspects, the adipose tissue is considered an innovative source of mesenchymal stem cells suitable for cell-based therapy in regenerative medicine [12]. The regenerative features of ASCs are attributable to their ability to secrete angiogenetic factors and immunomodulatory properties that facilitate tissue repair [5].

Increasing evidence shows that lipotransfer in sclerotic tissues may decrease collagen deposition and increase elasticity and vascularization [13]. To measure these changes, we used cutaneous resonance running time (CRRT), which is a noninvasive apparatus to assess skin biophysical properties [14]. CRRT can be influenced by the collagen content, skin elasticity, and hydration. CRRT is negatively correlated with skin stiffness or firmness. For instance, it has been demonstrated that CRRT is decreased in aged skin. In the aging process, defragmentation of the elastin network and configuration change in the dermal collagen network could increase skin stiffness and decrease skin elasticity and CRRT [14]. In our study, mean CRRT values increased after AFT, although the changes were not significant. Indeed, changes of cutaneous biophysical properties after fat transfer have not been well documented. It would be better if we could perform skin biopsy before and 3 months after the procedure to assess the possible histopathological changes in the pattern and content of collagen and elastic fibers. Unfortunately, none of the participants agreed to undergo skin biopsy. Following AFT in patients with scleroderma, a partial restoration of skin structures has been demonstrated by histological evaluation of the biopsies sections [11]. Del Papa et al. showed, by comparing with the baseline, a reduction in the dermoepidermal junction flattening with the reconstruction of the normal rete ridges and dermal papillae pattern in posttreatment samples [11].

In recent decades, autologous fat tissue grafting has been successfully used to regenerate atrophic or fibrotic skin for a large number of clinical conditions such as radio dermatitis, burning scars, linear scleroderma, and different types of morphea [8]. In most of these cases, a significant increase in skin elasticity and thickening with both aesthetic and functional improvement has been reported [8].

Moreover, the use of lipotransfer to reverse fibrosis is currently being explored in the treatment of the face and hands of patients with SSc [5, 15]. Del papa et al. treated perioral thickening in 20 female patients with the diffused type of SSc with autologous fat. After 3 months of the treatment, a significant increase was observed in the patients' maximum interincisive distance with respect to the baseline value (mean increase: 2.63 mm) [11]. Furthermore, they showed an increase in the neovascularization of the treated perioral skin after AFT [11]. Similarly, Sautereau et al. demonstrated improvements in mouth opening, facial pain, and MHISS scores in all the 14 SSc patients who were treated with autologous micro fat grafting at 3 and 6 months after surgery [16].

There are many ways to process fat after its collection; Del Papa et al. used Coleman's technique. They centrifuged lipoaspirate at 700 × g for 3 minutes before injection [11]. Whereas Sautereau et al. used the pure graft filtration technique to purify the lipoaspirate from blood cells and free lipid content [16]. There is no agreement among authors regarding the best method for processing fat transfer. Similar to our study, Onseti et al. used sedimentation by gravity as a method to eliminate nonviable components of the lipoaspirate. They compared the effects of lipotransfer and expanded 8 × 10^5^ cells/ml of the adipose stem cell injection in 10 patients with SSc. At the one-year follow-up, they noticed that both procedures provided a significant improvement in the mouth opening capacity and MHISS scores; but neither technique offered superior results [17].

In a recent trial, Virzi et al. demonstrated the beneficial effects of the combined use of autologous lipoaspirate and platelet-rich plasma (PRP) in the improvement of the buccal rhyme, skin elasticity, and vascularization of the perioral and malar areas of patients with SSc [18]. Whether this combination is superior to the standard fat transfer or other processing techniques in terms of clinical efficacy or durability needs to be addressed by prospective randomized clinical trials.

One limitation of the current and previous studies is the lack of quantified measurement of fat graft survivability. The actual mechanism on how fat graft survives is not completely understood [19]. According to previous studies, there was no significant difference in the survival of grafted fat obtained from different harvests and implantation techniques [20, 21]. Not any one technique is clearly superior to other techniques. There is no linear relation between the fat graft volume and survival rates [21]. Differences in underlying disease processes or patient variability may significantly impact engraftment. Significant differences in the number of stromal adipose stem cells in lipoaspirates between patients, and underlying conditions associated with poor revascularization, may account for the differences observed between patients in volume retention from fat grafts [20]. To determine if the injected fat in patients with scleroderma can be expected to survive as long as in the normal population, larger and controlled studies are required.

In the current study, all our participants were women because systemic sclerosis affects women three to four times as often as men [2], orofacial manifestations of scleroderma are four times more common in women [22], and men are generally poor consumers of aesthetic care and dermatology services. Autologous fat transfer has been successfully applied to men and even children [23] in different indications. Apart from fat availability which may be a limit in these groups, the aesthetic and functional results are comparable to women. In recently published articles [11, 15–18] on AFT for scleroderma, among a total of 57 cases, 4 cases were men, and the clinical outcomes were satisfactory, indicating the utility of the AFT in treatment of facial sclerosis in both females and males.

There are limited treatment options for scleroderma microstomia. The recommended treatment for limited mouth opening is based on stretching exercise for 3 months that need to be continued in the long term [4]. Intense pulsed dye light [24] and Co2 laser [25] have been advocated for the treatment of limited mouth opening; however, the reported efficacy was limited. There is a growing body of evidence that suggests autologous fat transfer can be an effective therapeutic alternative in patients with SSc. Our findings support the possible therapeutic role of autologous fat grafting in improving facial scleroderma both in aesthetic and functional aspects.

## Figures and Tables

**Figure 1:**
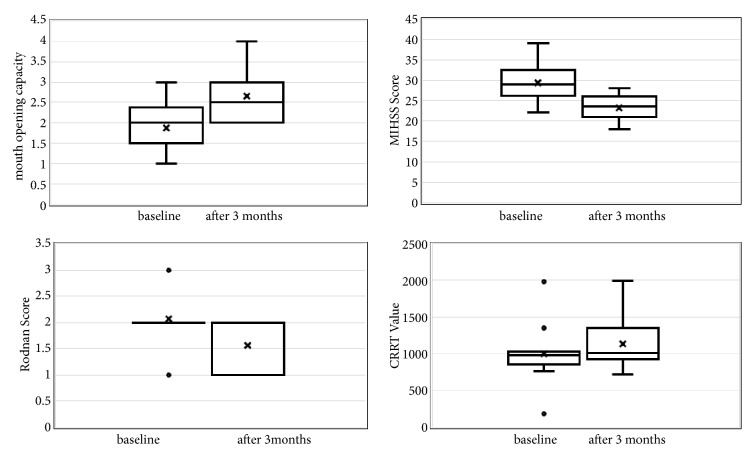
Pre- and postoperative changes of several parameters related to oral opening, mouth handicap in systemic sclerosis (MIHSS), sclerosis (Rodnan score), and cutaneous resonance running time (CRRT).

**Table 1:** The mouth handicap in systemic sclerosis scale (MHISS).

		Never	rarely	occasionally	often	always
1	I have difficulties opening my mouth	0	1	2	3	4

2	I have to avoid certain drinks ( sparkling, alcohol, acidic)	0	1	2	3	4

3	I have difficulties chewing	0	1	2	3	4

4	My dentist has difficulties taking care of my teeth	0	1	2	3	4

5	My dentition has become altered	0	1	2	3	4

6	My lips are retracted and/or my cheeks are sunken	0	1	2	3	4

7	My mouth is dry	0	1	2	3	4

8	I must drink often	0	1	2	3	4

9	My meals consist of what I can eat and not what I would like to eat	0	1	2	3	4

10	I have difficulties speaking clearly	0	1	2	3	4

11	The appearance of my face is modified	0	1	2	3	4

12	I have trouble with the way my face looks	0	1	2	3	4

**Table 2:** Baseline demographic, clinical, and therapeutic characteristics of patients.

Case	Age/sex	Disease duration (year)	Systemic sclerosis type	Liposuction area	Injected fat volume (ml)
1	41/f	8	limited	trochanteric	40

2	54/f	9	diffuse	Buttock	20

3	31/f	5	diffuse	flank	15

4	38/f	6	limited	periumbilical	30

5	37/f	7	diffuse	Buttock	20

6	32/f	5	diffuse	periumbilical	30

7	51/f	8	limited	trochanteric	25

8	49/f	10	limited	periumbilical	30

9	31/f	5	diffuse	trochanteric	20

10	34/f	8	diffuse	trochanteric	30

11	52/f	6	diffuse	Buttock	35

12	34/f	4	limited	trochanteric	40

13	29/f	5	diffuse	trochanteric	25

14	43/f	4	limited	periumbilical	30

15	39/f	7	diffuse	Buttock	15

16	32/f	7	diffuse	Buttock	30

## Data Availability

The data used to support the findings of this study are available from the corresponding author upon request.
